# Epidemiology of High Intensity Functional Training (HIFT) injuries in Brazil

**DOI:** 10.1186/s13018-022-03424-7

**Published:** 2022-12-05

**Authors:** Thiago T. Serafim, Nicola Maffulli, Filippo Migliorini, Rodrigo Okubo

**Affiliations:** 1 Physiotherapy Nucleus Orthopedic Trauma of Health and Sports Science of the Santa Catarina State (UDESC), Florianópolis, Brazil; 2 Department of Medicine, Surgery and Dentistry, University of Salerno, 84081 Baronissi, SA Italy; 3 School of Pharmacy and Bioengineering, Faculty of Medicine, Keele University, Stoke on Trent, ST4 7QB England; 4 Barts and the London School of Medicine and Dentistry, Centre for Sports and Exercise Medicine, Mile End Hospital, Queen Mary University of London, London, E1 4DG England; 5 Department of Orthopaedic, Trauma, and Reconstructive Surgery, RWTH University Hospital, Pauwelsstraße 30, 52074 Aachen, Germany

**Keywords:** High intensity functional training, Injury, Injuries, CrossFit, Resistance training

## Abstract

**Background:**

High intensity functional training (HIFT), usually called CrossFit, is a physical training that has gained much popularity in the past few years. The risk of acute and overuse injuries in HIFT is unclear. This study evaluated the incidence of injuries in HIFT, characterizing severity, location, and associated risk factors.

**Methods:**

This cross-sectional study was conducted between January and May 2021. HIFT practitioners were recruited through social media and answered an online questionnaire on training characteristics and injury history.

**Results:**

A total of 606 subjects (264 male and 342 female) were included. The average age of the participants was 29.78 ± 7.14 years. The mean height was 169.60 ± 8.96 cm, and the mean body mass was 73.69 ± 13.11 kg. Overall, participants were involved in HIFT for an average of 25.36 ± 20.29 months. A total of 58.6% of participants took part in 5 to 6 training sessions per week, 31.7% practiced 5 to 6 h per week. 62.7% of the responders performed other physical activities in parallel, 98.2% performed warm-up before the training, and a formal cooldown was accomplished by 29.4% of participants. 6.8% of athletes followed individual worksheets. 45.9% of participants participated in competition.

**Conclusions:**

The overall rate of injuries was 3.51/1000 h. 59.2% of subjects experienced two or more injuries. The shoulder was involved in 21.3% of cases, lower back in 18.3%, and the knee in 13.4%. No difference was found in injury rate between males and females. Experienced athletes were more prone to injury compared to those who trained under 12 months. Approximately the half of injuries did not cause training interruption. No difference was found in injury rate between males and females. The purpose of the participant did not impact the injury rate, nor did the practice of warm-up and cooldown, the time of weekly training, the league and level of competition. Finally, the participation in other sports in parallel did not demonstrated association with the injury occurrence.

*Trial registration*: The present study was approved by the Ethics and Research Committee by Plataforma Brazil and follows the Resolution 466/2012/CNS/MS/CONEP.

## Introduction

High intensity functional training (HIFT) has become popular over the years [1]. HIFT is believed to promote greater physical and health benefits than other less intensive traditional modalities [1, 2]. CrossFit is a well-known modality of HIFT [3]. HIFT involves several exercises in different areas which includes multiple movements of Olympic gymnastics (movements with bodyweight), Olympic weight lifting (squats, throws, presses, deadlifts), and metabolic conditioning (alactic, lactic and aerobic). This training method is characterized by performing functional exercises constantly varied and at high intensity [4]. HIFT requires challenging and motivational character of participants, which makes the level of engagement high within the sport [5]. Associated with this, the competitive factor also increases adherence, but this can be a contributor to the increase in injuries [6]. With this information, the American College of Sports Medicine (ACMS) classified it as high risk of injury [6–8], though HIFT resulted in similar rate of injuries compared to other popular sports (e.g., football, rugby, handball, running) [4, 9]. Previous investigations reported that the incidence of injury among different modalities of HIFT ranged from 1.94 to 3.4 events per 1000 h of training [10–13]. In the Brazilian population, previous epidemiological investigations found an incidence ranging from 0.80 to 18.9 per 1000 h of exposure [11, 14]. The reason behind this wide range may be the heterogeneous modalities of HIFT and participants included. Deeper knowledge on injury patterns and risk factors is of great importance to prevent acute and overuse traumas and to improve training efficacy.

The present cross-sectional study investigated the rate, location, and severity of injuries among HIFT practitioners. Risk factors that may predispose to injury were also investigated.

## Material and methods

### Study design

This retrospective cross-sectional study followed the Strengthening the Reporting of Observational studies in Epidemiology (STROBE) guidelines [15]. The present study was approved by the Ethics and Research Committee by Plataforma Brasil and follows the Resolution 466/2012/CNS/MS/CONEP. All individuals were volunteers who were informed about the research objectives, data confidentiality, and signed the Informed Consent Form.

### Participant recruitment

Participants were recruited through social media, with formal invitations posted on Instagram (Meta Platforms, US). This Instagram post was titled "CrossFit Injuries", explaining that it was a master's research questionnaire with a link and average time spent to answer (5 min) and who could participate (CrossFit practitioners, regardless of practice time). Participants were HIFT practitioners from Brazil. HIFT was defined as a “training style that incorporates functional, multimodal movements, performed at relatively high intensity, and designed to improve parameters of general physical fitness and performance.” [16]. Only practitioners older than 18 years were considered. Only participants who answered all the questions and filled them out correctly and completely were considered. For eligibility criteria, the PICO method was used [17]:

*P (Population):* practitioners over 18 years of age.

*I (Intervention):* High Intensity Functional Training.

*C (Comparison):* Non-HIFT Practitioners.

*O (Outcome):* Injuries rate, modality, level, and timing of training, complementary activities.

### Data collection

Between January and May 2021, participants could access the Google Forms Tool posted on Instagram to access the questionnaire (https://forms.gle/TwWwovbw714onihu8). The questionnaire was developed and evaluated by experts, and was composed of 20 questions about demographics, level and type of training (sessions per week and hours per week, differing the daily training load), participation in other sport activities, previous injuries or instructor supervision. Injury was clearly defined in the questionnaire to avoid misinterpretation. Injury was defined as a physical damage to a part of the body that caused the loss or modification of one or more training sessions, or hindered daily activities [18]. The severity of an injury was rated according to the period of days off activities: trivial (0 days), minimal (1–3 days), mild (4–7 days), moderate (8–28 days) or severe (> 28 days) [19]. The experience level was divided according to the time of experience into beginners (0–5 months), intermediate (6–11 months) and experienced (≥ 12 months). Also, questions about performing or not of warm-up and cooldown, which were, respectively, defined as a set of light exercises with stretching and joint mobilization performed to prepare the body for more vigorous exercises, and cooldown as a set of exercises that gradually decrease the intensity and promote a return to a state close to rest after training. Furthermore, data on the treatment of injuries and instructions on training were collected.

### Data analysis

The statistical analyses were conducted using the IBM SPSS Software (version 20.0). Confidence intervals were set at 95%. Values of *p* < 0.05 were considered statistically significant. To calculate the total incidence of injuries per 1000 h, the model proposed by Minghelli et al. 2019 was used [20]. The sum of injuries of the individuals studied was multiplied by the total number of hours of training of the injured individuals. Of this total value, the proportion to 1000 h was performed. To calculate hours, the number of 7 h per week and 1 month = 4.34 weeks was used. Also, the injury rate per group of practitioners (beginners, intermediate, experienced) was calculated by dividing the number of injuries per group by the time in months of practice experience for each group.

## Results

### Characterization of the demographic distribution

**Fig. 1:**
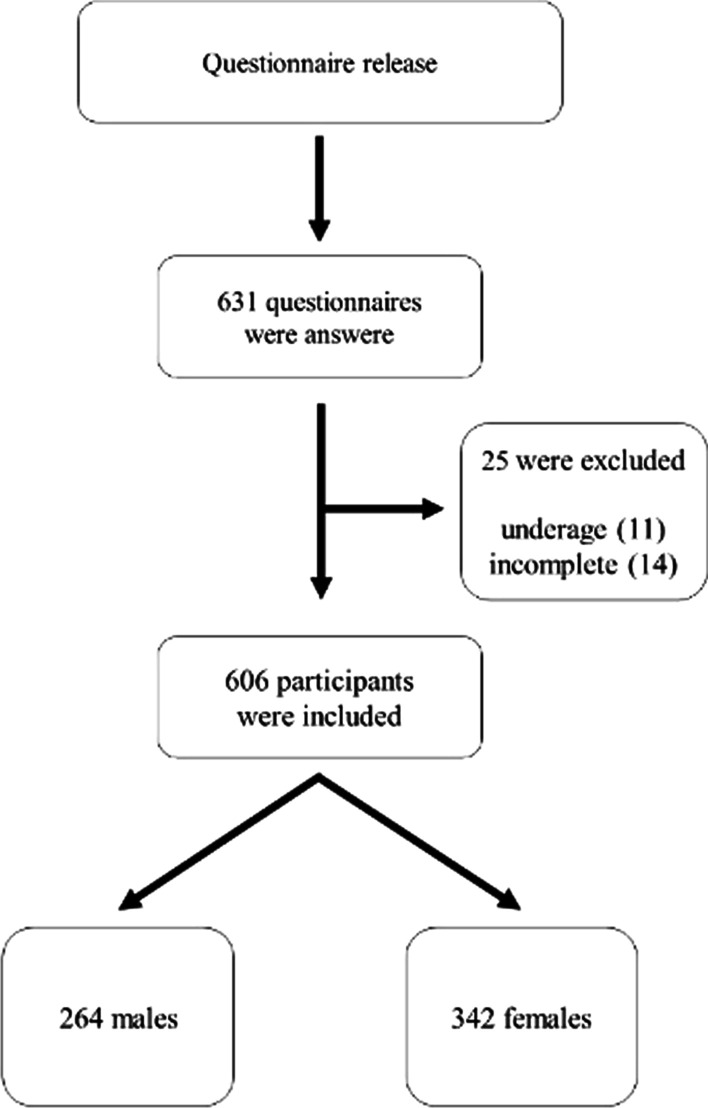
Participant recruitment

A total of 631 questionnaires were answered, but 25 were excluded for not meeting the eligibility criteria: underage (*N* = 11), incomplete questionnaires (*N* = 14). Thus, 606 participants were included (Fig. 1). There were 264 males and 342 females. The average age of the participants was 29.8 ± 7.14 years, and the mean BMI was 25.3 kg/m^2^.

### Training characteristics

Overall, participants were involved in HIFT for an average of 25.36 ± 20.29 months. A total of 58.6% (355 of 606) of participants took part in 5 to 6 training sessions per week, and 31.7% (192 of 606) practiced 5 to 6 h per week. 40.8% (247 of 606) of participants performed HIFT to promote their physical health, 27.4% (166 of 606) to improve their physical performance, 15.2% (92 of 606) to lose weight, 6.3% (38 of 606) to improve hypertrophy, and 4.3% (26 of 606) as physical conditioning. 62.7% (380 of 606) of the responders performed other physical activity in parallel, 98.2% (595 of 606) performed warm-up before the training, and a formal cooldown was performed by 29.4% (178 of 606) of participants.

93.2% (565 of 606) of participants followed the conventional classroom model as training, and 6.8% (41 of 606) had individual worksheets. 45.9% (278 of 606) of participants participated in competition. The league among participants involved in competition was local in 71.8% (196 of 273), 20.5% (56 of 273) regional, 5.9% (16 of 273) national, and international 1.8% (5 of 273). The category in which participants competed was beginner in 50% (135 of 270), intermediate in 34.1% (92 of 270), and advanced in 15.9% (43 of 270). The average of instructors who supervised practitioners per training session ranged from 1.7 to 10.6. Participant demographic is shown in Table 1.

**Table 1:** Participant demographic

Endpoint	*n* (%)
Age	29.78 ± 7.1
Height (cm)	169.60 ± 8.9
Body weight (Kg)	73.7 ± 13.1
Goal with training	*n*
Health	247 (40.8%)
Performance	166 (27.4%)
Conditioning	26 (4.3%)
Slimming	92 (15.2%)
Hypertrophy	38 (6.3%)
Other goal	37 (6.1%)
Weekly frequency	*n*
1–2 days	24 (4.0%)
3–4 days	209 (34.5%)
5–6 days	355 (58.9%)
> 7 days	18 (3.0%)
Week hours of training	*n*
1-2 h	186 (30.7%)
3-4 h	133 22.0%)
5-6 h	192 (31.7%)
7-10 h	75 (12.4%)
> 11 h	20 (3.3%)
Warm-up	*n*
Yes	595 (98.2%)
No	11 (1.8%)
Cooldown	*n*
Yes	178 (29.4%)
No	428 (70.6%)
Training method	*n*
Collective class	565 (93.2%)
Individual training	41 (6.8%)
Parallel exercise	*n*
Yes	380 (62.7%)
No	226 (37.0%)
Competition	*n*
Yes	278 (45.9%)
No	328 (54.1%)

### Characteristics of injuries

A total of 644 injuries were reported. The overall incidence of injuries was 3.51 per 1000 h of exposure. 40.8% (247 of 606) of participants did not experience injuries, 59.2% (359 of 606) experienced at least one injury. Of them, 45.9% (165 of 359) experienced two or more injuries, and 54.1% (194 of 359) only one. 21.3% (138 of 644) of injuries were in the shoulder, 18.3% (118 of 644) in the lower back, 13.4% (86 of 644) in the knee, and 10.2% (66 of 644) in the wrist (Table 2).

**Table 2:** Injury locations

Area of the body	*n* (%)
Shoulder	138 (21.4%)
Low back	118 (18.3%)
Knee	86 (13.4%)
Wrist	66 (10.2%)
Ankle	29 (4.5%)
Elbow	27 (4.2%)
Back	25 (3.9%)
Hip	23 (3.6%)
Neck	22 (3.4%)
Hand	18 (2.8%)
Leg	18 (2.8%)
Foot	8 (1.2%)
Thigh	16 (2.5%)
Head	4 (0.6%)
Other and not specified	46 (7.1%)

56.3% (363 of 644) of injuries did not cause training interruption. Of the total number of injuries, 9.5% (61 of 644) were classified as minimal, 12.3% (79 of 644) as mild, 10.9% (70 of 644) as moderate, 11.1% (71 of 644) as severe. 54.9% (354 of 644) of injuries were managed by health care personnel, 37.6% (242 of 644) by the participant themselves, 7.5% (48 of 644) did not require any treatment.

### Associated factors

Associated factors are shown in greater detail in Table 3. No difference was found in injury rate between males and females (*p* = 0.07). Experienced athletes were less prone to injury compared to those who had trained for less than 12 months (*p* = 0.001). The purpose of the participant did not impact the injury rate (*p* = 0.5), nor the presence of warm-up or not (*p* = 0.8) and cooldown (*p* = 0.06). The time of weekly training did not show any association with the injury rate (*p* = 0.5). The league and level of competition did not influence the injury rate (*p* = 0.1 and 0.2, respectively), nor the involvement of participants in competition (*p* = 0.2). The participation in other sports in parallel to HIFT did not demonstrate association with injury occurrence (*p* = 0.6).

**Table 3:** Analysis of the factors associated with the injury

Variable	Events	Rate (injury/month)	Total	*p*
Gender				
Male	167 (46.5%)	−	359 (100%)	0.07
Female	192 (53.5%)			
Experience level				
Beginner	31 (8.6%)	9.3	359 (100%)	0.001*
Intermediary	61 (17.0%)	8.12		
Experienced	267(74.4%)	6.29		
Training objective				
Health	159 (44.3%)	−	359 (100%)	0.3
Performance	102 (28.4%)			
Conditioning	16 (4.5%)			
Slimming	47 (13.1%)			
Hypertrophy	15 (4.2%)			
Other	20 (5.6%)			
Weekly volume				
1–2 h	110 (30.6%)	−	359 (100%)	0.5
3–4 h	70 (19.5%)			
5–6 h	120 (33.4%)			
7–10 h	47 (13.1%)			
≥ 11 h	12 (3.3%)			
Warm-up				
Yes	352 (98.1%)	−	359 (100%)	0.8
No	7 (1.9%)			
Cooldown				
Yes	95 (26.5%)	−	359 (100%)	0.05
No	264 (73.5%)			
Training method				
Collective	331 (92.2%)	−	359 (100%)	0.2
Individual	28 (7.8%)			
Competition				
Yes	173 (48.2%)	−	359 (100%)	0.2
No	186 (51.8%			
Competition league				
Local	114 (67.5%)	−	169 (100%)	0.1
Regional	39 (23.1%)			
National	11 (6.5%)			
International	5 (3.0%)			
Competition level				
Beginner	82 (49.1%)	−	167 (100%)	0.5
Intermediary	5 (32.9%)			
Advanced	30 (18.0%)			
Other sports in parallel				
Yes	228 (63.5%)	−	359 (100%)	0.6
No	131 (36.5%)			

*p < 0.05

## Discussion

This study aimed to investigate the rate, location and severity of injuries among HIFT practitioners. Experienced athletes were less prone to injury compared to those who trained for less than 12 months. The shoulder was the area of the body at higher risk of injuries in HIFT Brazilian practitioners, followed by lower back and knee. Approximately half of injuries did not cause training interruption. No difference was found in injury rate between males and females. The purpose of the participants did not impact the injury rate, nor the presence of warm-up and cooldown, the time of weekly training, the league and level of competition. Finally, the participation in other sports in parallel of HIFT did not demonstrated association with injury occurrence.

### Epidemiological values

The rate of injuries in the present study (3.51/1000 h) agreed with those found in previous reports [21–23]. This incidence is comparable to other more traditional resistance training modalities, such as weightlifting and powerlifting [24, 25]. This study demonstrated that 59.2% of HIFT practitioners experienced an injury. This prevalence is similar to previous reports [21, 26, 27]. In a previous online questionnaire epidemiological investigation, Hak et al. [28] found an injury rate of 73.5%. Teixeira et al. [29] and Toledo et al. [30] found a prevalence of injury of 38.6% and 38.5%, respectively. However, the authors performed the evaluation in a face-to-face fashion. The online questionnaire may be biased toward the research targeted public, which can draw more attention by who already had an injury. Moreover, there are some variations between definitions of injuries between studies [3, 22, 23, 31–33].

### Anatomical features

More than 20% of the injuries reported in the present study were located in the shoulder, followed by the lower back, and the knee. These findings agreed with those observed by previous studies [9, 18, 34]. Shoulder injuries are common in sports, especially in overhead athletes [34]. Olympic weightlifting exercises and gymnastics have high shoulder demands, with large ranges of motion and high loads [4]. Some exercises are introduced in the warm-up to avoid such injuries, but the method needs to be better defined [34]. Likewise, authors suggested that combined range of motion (ROM) and stability exercises should be introduced in the complementary training to prevent shoulder injuries [35, 36]. High loads also affect the lumbar region, especially in exercises such as deadlift and back squat [4, 37]. These require an adequate lumbopelvic control for the performance and optimal ROM in the lower limbs [38]. In addition, the pursuit of a personal best can increase the chances of injury because of a sudden increase in load [39]. HIFT requires constant movements which may affect the knee [4]. Reduced ROM in the ankle [40] and weakness of the hamstrings [41] may increase the risk of knee injuries. A stiff ankle produces greater knee overload, as well as a low agonist/antagonist ratio between quadriceps and hamstrings [40]. Squats involve the lower limbs in a closed kinetic chain, but with a much higher proportion of quadriceps activation compared to hamstrings. This further favors the difference in strength between the two muscle groups, increasing the chances of injury [40]. Thus, the implementation of specific exercises for hamstrings is important in reducing the agonist/antagonist strength difference in the knee [42, 43].

### Associated factors

The only variable that was associated with injury was practice time. Individuals with more experience experienced more injuries (in absolute numbers) compared to beginners participant. However, when we look at the monthly injury rate, we see that beginners have an increased ratio. In line with these results, other studies have also found an association between the time of practice in CrossFit and the presence of injuries [3, 9, 29]. Szeles et al. [11] found no association between more experienced individuals and increased injury. Mehrab et al. [18] that the risk of injury was significantly increased in athletes participating in CrossFit for less than 6 months, suggesting that coaches and athletes should focus on correct movement patterns and scale workouts for beginners. If beginners do not scale or scale incorrectly, they may be more prone to injuries for overuse and the complexity of the workouts and movements, as we found that the majority of injuries were chronic/overuse.

In contrast, not all studies agree with this Alekseyev et al. [32] compared the injury rates of experienced, intermediate and beginners practitioners. These authors found that experienced individuals are 2.3 times and 7.2 times more likely to get injured than other subjects, respectively. These results demonstrate that the chances of injury are time-dependent. Feito et al. [9] also found similar results. Furthermore, Teixeira et al. [29] found that more experienced individuals (> 2 years of practice) have 3.7 times more chances of injury than beginners (< 6 months of practice).

In line with these results, another study conducted in Brazil and a questionnaire survey of 566 individuals showed that individuals who have been training longer (more than six months) had a significantly higher incidence of injuries than individuals who were beginners (less than six months) [44]. The injury rate, despite decreasing, according to our results, is still considerable during the performance and time of practice of this modality. Some authors explained by this type of effort experienced professionals perform. Even improving technique, these experienced athletes usually use larger loads and face more extreme situations, being able to work with greater volume and intensity, and consequently with shorter recovery time. This leads to a greater predisposition to injuries, or “greater exposure equates to more chances in which injury can occur” [45].

### Limitations

This study certainly has limitations. As this is a retrospective cross-sectional study, participants may have forgotten an injury. In addition, the online questionnaire may have biased selection, as individuals who have been injured at some point may have a greater predisposition to complete the survey. Although the survey was advertised throughout Brazil, there were not responses from all states, and there was a greater concentration in the south and southeast. When it comes to studies on injuries at HIFT, there is still difficulty in accessing practitioners in gyms, as there is some denial on the part of owners and teachers. It is necessary to understand that research should be seen as an ally for the improvement of the sport and not as an enemy.

### Practical implications

The results are related to the majority of studies already carried out in this format. Thus, the consistency of the results demonstrates relevance. As it is a physical conditioning program, HIFT should be introduced as safely as possible. This is a challenge in many disciplines and it is no different in the HIFT setting. Understanding the main injuries in HIFT helps training centers to intervene to reduce the number of injuries and is the first step to be analyzed. Also, when there is information about the types of injuries and risk factors for this, there is an even greater chance of trying to reduce this number (second step). As a third step to injury prevention, assessment is important. From the moment an individual starts training, an assessment (with medical history and physical examination) is ideal. This more specifically directs what the beginner needs to work on. Based on the sporting context and the evaluation carried out, the fourth step is the performance of complementary exercises, how ROM, stability and strength exercises.

## Conclusion

The overall rate of injuries was 3.51/1000 h. 59.2% of subjects experienced two or more injuries. The shoulder was involved in 21.3% of cases, the lower back in 18.3%, and the knee in 13.4%. No difference was found in injury rate in between males and females. Experienced athletes were more prone to injury compared to those who had trained for less than 12 months. Approximately half of injuries did not cause training interruption. The purpose of the participant did not impact the injury rate, nor did the presence of warm-up and cooldown, the time of weekly training, the league and level of competition. Finally, participation in other sports in parallel did not demonstrated association with injury occurrence in HIFT.

## Data Availability

The data that support the findings of this study are available from Thiago Teixeira Serafim but restrictions apply to the availability of these data, which were used under license for the current study, and so are not publicly available. Data are however available from the authors upon reasonable request and with permission of Thiago Teixeira Serafim via e-mail.

## Abbreviations

HIFT: High intensity functional training
ACMS: American College of Sports Medicine
STROBE: Strengthening the Reporting of Observational studies in Epidemiology
US: United States
PICO: Population, Intervention, Comparison, Outcome
ROM: Range of Motion
